# Horseshoe appendix: A case report and literature review

**DOI:** 10.1097/MD.0000000000041425

**Published:** 2025-02-28

**Authors:** Hannah Pflieger, Christine Tchikladzé-mérand

**Affiliations:** a Visceral and digestive surgeon, Hôpital Privé Guillaume de Varye, ELSAN, Saint-Doulchard, France; b Biostatistician, ELSAN, Paris, France

**Keywords:** acute appendicitis, case report, duplex appendix, emergency surgery, horseshoe appendix

## Abstract

**Rationale:**

Horseshoe appendix is 1 of the rarest types of duplex appendix characterized by 1 appendix having 2 openings at the cecum. Clinically, it is extremely rare and is detected incidentally at surgery. Therefore, accurate and timely diagnosis is important for enhanced prognosis, treatment planning, and optimizing patient outcomes. This case report presents the first case of horseshoe appendix in France.

**Patient concerns:**

A French 15-year-old boy, presented to the emergency department with pain in the right iliac fossa that had been worsening for 4 days. Considering his symptoms, abdominal ultrasound, radiological examination, and computed tomography (CT) scan results, an acute appendix was suspected.

**Diagnoses:**

Acute appendicitis (AA) with “horseshoe” type appendix.

**Interventions:**

Surgical intervention with exploratory laparoscopy was performed immediately.

**Outcomes:**

We found a heterogeneous image, partially fluid in the right iliac fossa, measuring approximately 16 × 9 mm, with inflammatory remodeling of the fat upon contact. The radiological examination concluded that complicated AA was suspected. Finally, the pathological report revealed acute suppurative appendicitis with peritoneal reaction.

**Lessons:**

Horseshoe appendix is rare. Considering his symptoms, radiological examination, ultrasound, and CT scan results, an acute appendix was suspected. The patient was successfully treated using emergency surgery and antibiotics treatment thanks to further diagnosis via exploratory laparoscopy and pus microbial analysis.

## 1. Introduction

Acute appendicitis (AA) is the most common abdominal surgical emergency throughout the world, with an annual incidence of 96.5 to 100 cases per 100,000 adults.^[1]^ Although imaging such as computed tomography (CT) and magnetic resonance imaging has been known to be fundamental in the diagnosis of AA,^[2]^ laparoscopic appendectomy has become the standard treatment since the 1980s, as it is associated with less postoperative pain and a quicker recovery.^[1]^ Duplex appendix is rare type of appendiceal anomaly with a reported incidence of approximately 0.004% and has significant clinical and surgical importance.^[3]^ It was first discovered in 1867 by Bartles in fetal studies, and then observed in 1892 by Picoli in adult studies.^[4]^ The majority of duplex appendices are detected at surgery or postmortem examinations. Even though these are usually asymptomatic, typical symptoms may present with inflammation or obstruction of the organ. Frequently, episodes of abdominal pain have been previously reported.^[5]^ The most commonly used classification system for duplex appendix is the Cave–Wallbridge classification, originally containing types A, B1, B2, and C, but recently updated to include other subtypes like the horseshoe appendix (Table 1).^[6]^ According to Cave–Wallbridge classification, horseshoe appendix is referred as “Type D,” where both ends of the appendix communicate with the cecum with 2 separate stumps or bases located at a frontal or a sagital disposal.^[7]^ Interestingly, a few theories have been proposed on how horseshoe appendices can develop. So far, the most prominent 1 suggests that the appendix’s base splits during embryologic period due to an abnormality, which leads to separation of cecal growth and thus resulting in a double-based, yet single structure.^[8,9]^

**Table 1:**
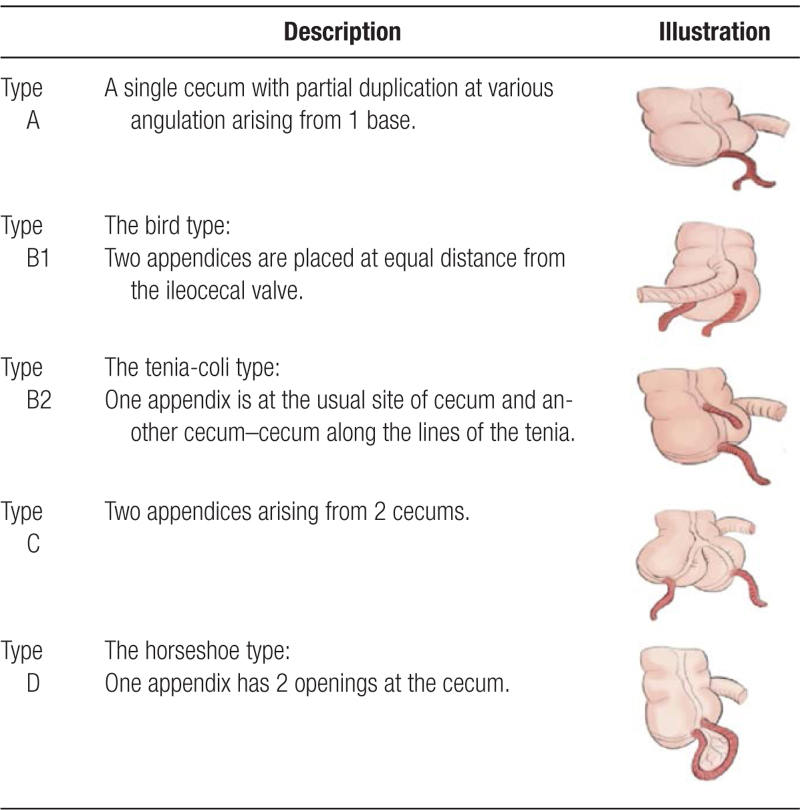
Cave–Wallbridge classification system for duplex appendix.^[6]^

In our case study, a 15-year-old boy, presented to the emergency department in January 2023 with worsening pain in the right iliac fossa. By combining his symptoms, abdominal ultrasound, radiological examination, and CT scan results, AA was considered. Emergency surgery was performed and to our knowledge, the first case of horseshoe appendix in France was discovered. This clinically rare form of duplex appendicitis may lead to poor clinical outcomes as well as serious health consequences, if misdiagnosed and mismanaged. However, there is no consensus on how it should be diagnosed or treated.

## 2. Case presentation

The case study was approved by the ELSAN clinic Institutional Review Board (IRB), and the patient gave his consent for using his clinical data for this case report.

### 2.1. Chief complaints

A 15-year-old child presented in the emergency department with pain in the right iliac fossa that had been worsening for 4 days. No nausea or vomiting was reported. He presented with chills, but no authenticated fever. Gastrointestinal transit was slowed but preserved.

### 2.2. History of past illness

The patient had no significant family history.

### 2.3. Physical examination

The patient was 1.80 m tall and weighed 54 kg, resulting in a body mass index of 16.67 kg/m². Clinical examination showed apyrexia, haemodynamic stability, as well as positive Blumberg and Psoas’ signs.

### 2.4. Laboratory examinations

Laboratory tests revealed a biological inflammatory syndrome, with hyperleukocytosis (leukocytes 12.8 G/L) and CRP levels increased to 113 mg/L.

### 2.5. Imaging examinations

**Figure 1.**
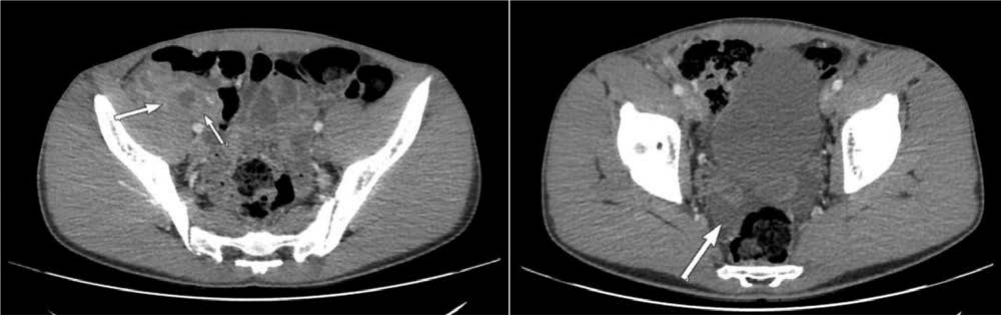
Abdomino-pelvic CT scan showing no significant abnormality of the last ileal loop. Several peri-centimeter lymph nodes were noted in the right iliac fossa, with a layer of fluid effusion. CT = computed tomography.

**Figure 2.**
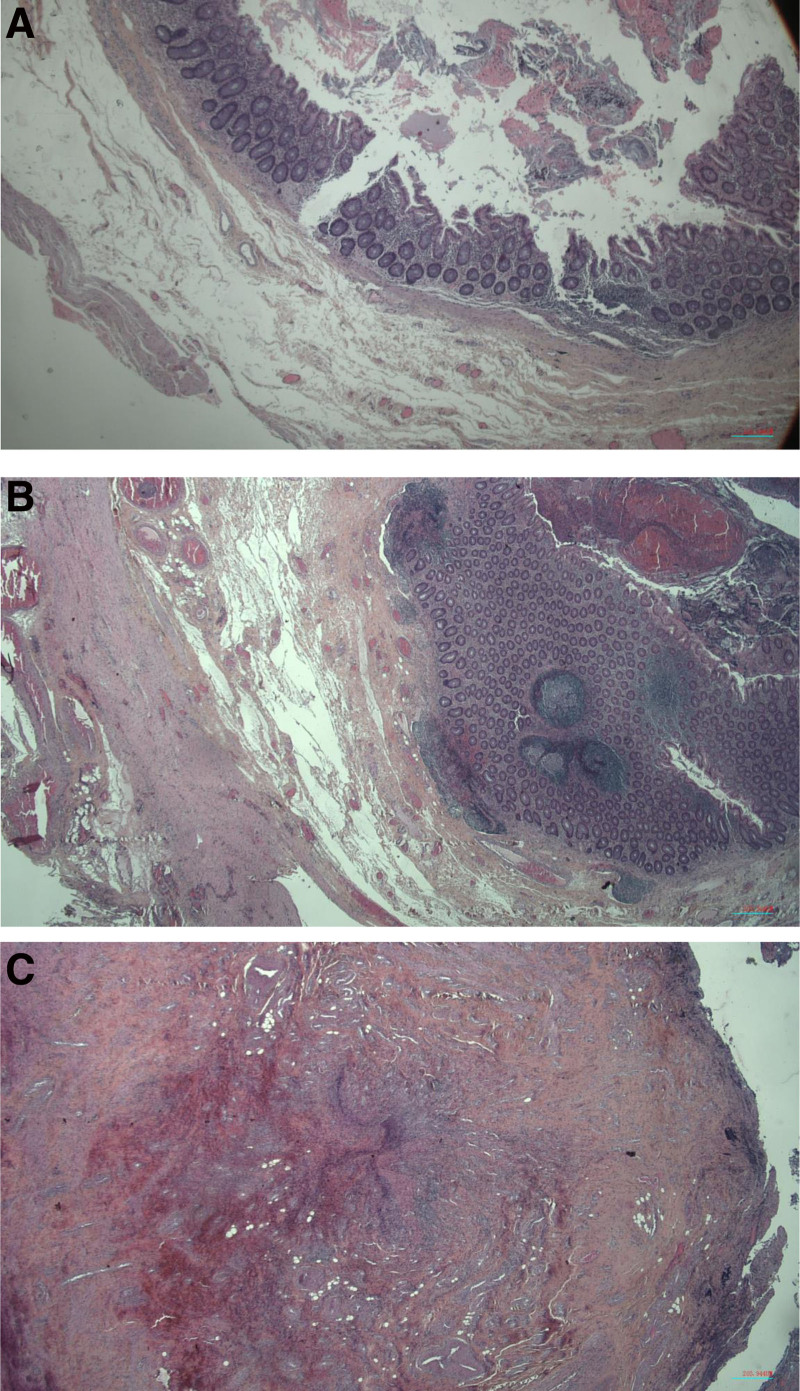
(A) Lesions of AA, with ulceration of the mucosa and a polymorphous inflammatory infiltrate extending throughout the wall. Fibrinoleukoyte coatings are also observed on the serosa, indicating peritonitis. (B and C) The edges of the stapled exegesis were not inflamed, and all the tunics in the appendix show a normal development. AA = acute appendicitis.

Abdominal ultrasound, supplemented by an abdomino-pelvic CT scan with injection, showed no significant abnormality of the last ileal loop. Several peri-centimeter lymph nodes were noted in the right iliac fossa, with a layer of fluid effusion (Fig. 1). The appendix was not individualized. We found a heterogeneous image, partially fluid in the right iliac fossa, measuring approximately 16 × 9 mm, with inflammatory remodeling of the fat upon contact. Microscopy histological images (Fig. 2) revealed lesions of AA, showing an ulceration of the mucosa and a polymorphous inflammatory infiltrate extending throughout the wall, as well as fibrinoleukoyte coatings on the serosa, which indicates a peritoneal reaction. Nevertheless, the edges of the stapled exegesis were not enflamed, and all the tunics in the appendix showed a normal development. The radiological examination concluded that complicated AA was suspected.

### 2.6. Treatment

**Figure 3.**
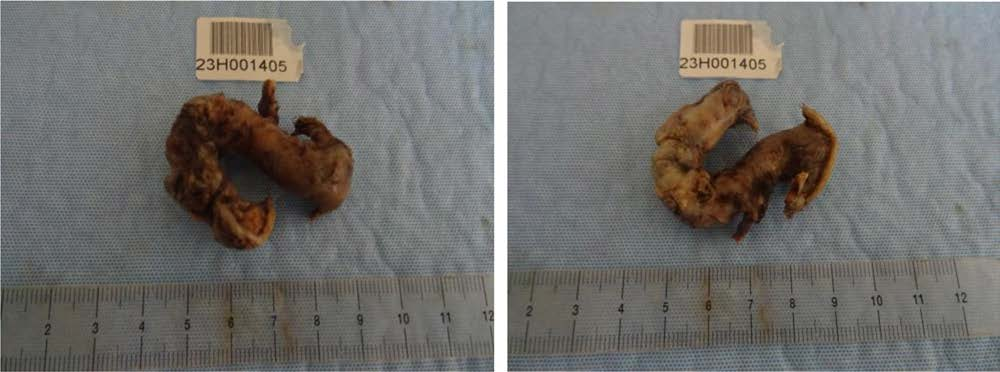
Inflamed appendix having 2 openings at the cecum showing a horseshoe-like appearance.

Surgical exploration using laparoscopy was carried out the same day. This revealed a “horseshoe” appearance of the appendix (Fig. 3), with an appendicular perforation at the medial level, with pus upon contact, of which a sample was taken and sent for bacteriological examination. The 5 mm trocar positioned in the right iliac fossa was extended to 12 mm in order to be able to insert the purple 45 mm endo-GIA stapler. This made it possible to staple the cecal basin, which allowed visualizing the sealing on the 2 legs of the appendix. A Blake Ch 19 drain was left in contact with the stapling zone, in a sloping position.

### 2.7. Bacteriological examination and antibiotic therapy

Treatment with amoxicillin + clavulanic acid (dose of 3 g/d) was initiated on day 0. The progress was rapidly favorable, enabling the drain to be removed on day 3. Bacteriological analysis of pus sample taken from the perforated appendix during surgery revealed *K oxytoca* resistant to amoxicillin + clavulanic acid. Antibiotic therapy was therefore changed on day 3 to ofloxacin (200 mg morning and evening) and metronidazole (500 mg × 3/d) for a total of 9 days.

### 2.8. Outcome and follow-up

The study’s outcomes demonstrate a successful case of AA management. The pathological report confirmed acute suppurative appendicitis with peritoneal reaction, indicating advanced stage of inflammation. The patient’s recovery was uneventful, which is a positive outcome following appendectomy. Discharge on day 4 is consistent with typical recovery timelines for appendicitis patients. The patient’s satisfaction with both medical and surgical management suggests that the treatment approach was effective and met his expectations. This outcome is important, as timely diagnosis, appropriate use of antibiotics, as well as surgical exploration and intervention was crucial for preventing complications such as perforation or abscess formation, which could have prolonged the recovery period and increased the risk of postoperative complications.

## 3. Discussion

Acute appendicitis is the most common abdominal emergency requiring surgery. Yet, most patients with appendicitis display a wide range of clinical manifestations including abdominal pain, making hard for physicians to diagnose it.^[10]^ Therefore, proper and timely clinical detection of AA is crucial due to high risk of potential perforation. Duplex appendix is rare type of appendiceal anomaly that occurs 1/25,000 of the cases.^[3]^ Its preoperative diagnosis is challenging as it is practically always identified intraoperatively, specifically via laparoscopy as it grants better visualization of the abdominal cavity.^[11]^

### 3.1. First case in France

Our case reported above falls under category D (horseshoe type) of the Cave–Wallbridge classification as there were 2 openings at the cecum of the appendix, as confirmed by laparoscopy. Abdominal ultrasound and abdomino-pelvic CT scan showed no significant abnormality of the last ileal loop. This misdiagnosis by preoperative radiological examinations was also observed in many similar cases of duplex appendicitis^[4,12,13]^ and a horseshoe type case.^[14]^ In clinical practice, physicians should consider the diagnosis in conjunction with the patient symptoms as well as radiological examinations and should not exclude the diagnosis of duplex appendicitis. If not considered, the time for diagnosis and adapted treatment could be missed, which may lead to serious complications, such as intraoperative failure to detect the second appendix that could get inflamed in the future.^[15]^ In our case, only 1 appendix with 2 openings at the cecum is affected. However, there was an appendicular perforation at the medial level, with pus upon contact. This was also observed in a similar case involving an adult patient who, on the other hand, experienced nausea and fever but her complete blood count and urine analysis revealed normal findings.^[4]^ This could be due to biological differences between adult and pediatric appendicitis such as the prevalence of multisystem inflammatory syndrome (MIS) in children with AA.^[16]^ Nevertheless, our patient did not present MIS as it is typically characterized by an inflammation with fever, rash, conjunctivitis, impaired coagulation, and gastrointestinal symptoms.^[17]^ Moreover, the bacteriological evaluation of the pus sample was shown to be advantageous in the nonoperative management of AA as it revealed *K oxytoca* resistant to initially proposed antibiotics treatment. A similar case of *K oxytoca* was reported in an 11-year-old immunocompetent boy who experienced nonperforated appendicitis, but a rare case of septicemia.^[18]^ To our knowledge, bacterial translocation most often affects the elderly. Lastly, it is important to acknowledge that our case report presents clinical findings of a single patient and does not represent the whole population of patients with horseshoe appendix. To address this limitation, we have incorporated a concise review summarizing all the existing cases reports. This review aims to explore patient demographics, clinical presentations, diagnostic approaches, and treatments employed for this rare condition with very low occurrence. If circumstances allow, conducting a multicenter retrospective study on horseshoe appendix in near future would be highly beneficial. Such a study could document past occurrences of this condition in appendiceal surgeries by examining hospital records, thereby enhancing our understanding of this unique anatomical variation.

Despite the above considerations, we detected and removed the horseshoe type appendix leading to an uneventful recovery. Follow-up of conservative management of appendicitis must be considered as chances of recurrence are high in patients who have been operated for duplex appendix removal. Interestingly, according to a case of a 17-year-old male patient with a surgical history of a previous appendectomy at 11 years of age, inspection of the cecum revealed a second, ruptured appendix with type B2 duplication. It had a discrete cecal origin and was located about 6 cm distal to the stump of the previously removed appendix.^[19]^ Therefore, it is crucial for visceral surgeons to be aware of appendiceal anomalies such as duplication and perform complete inspection of the cecum during an appendectomy.

### 3.2. Patient demographics

**Figure 4.**
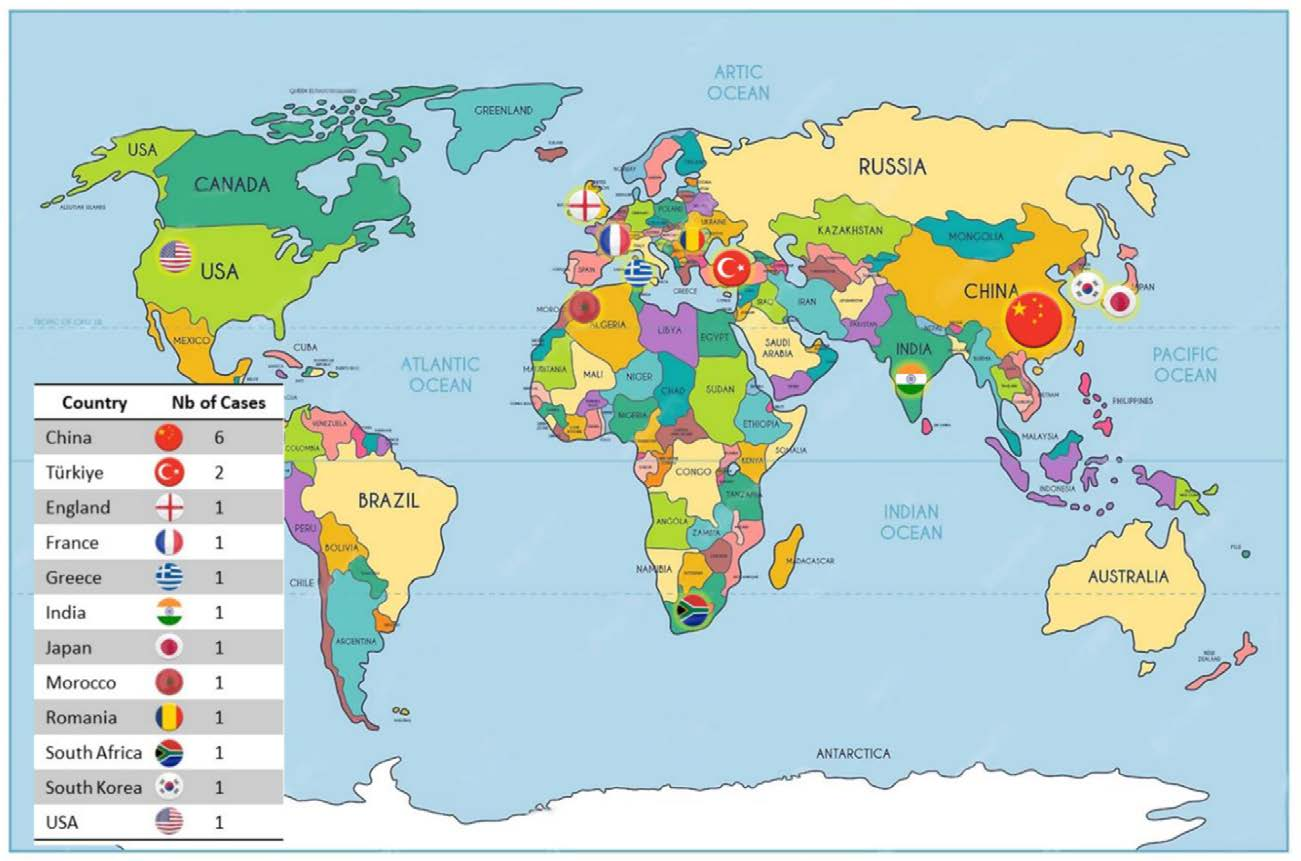
World’s locations of distinct cases of horseshoe appendix.

**Figure 5.**
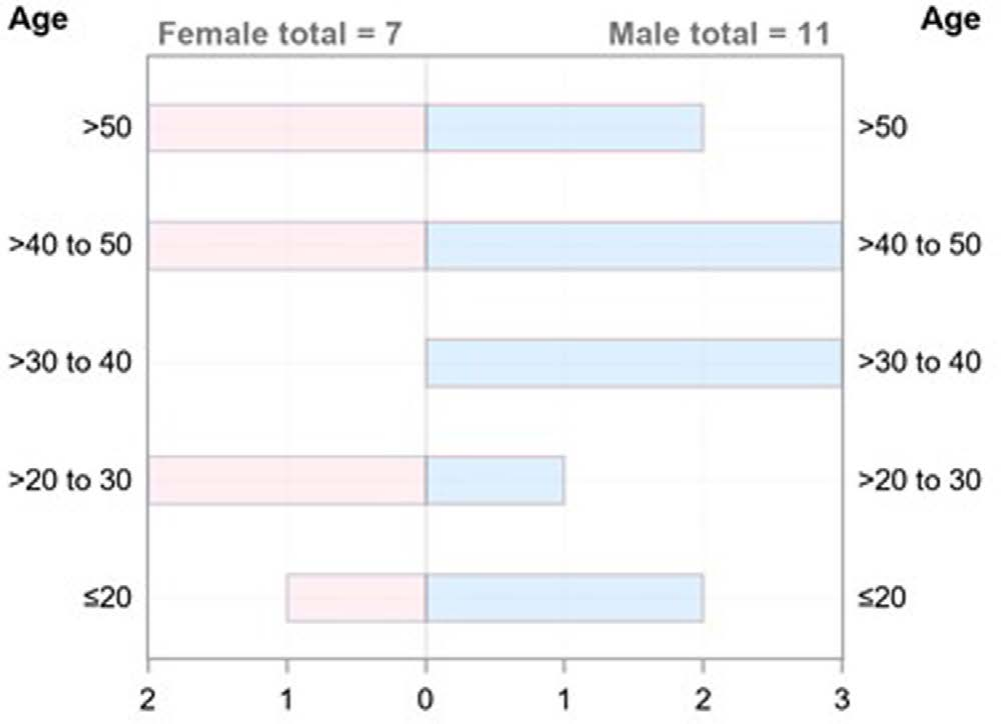
Graphical representation of age classes by sex in horseshoe appendix cases. The female:male ratio was 0.6, in which 61.1% of affected patients were males and 38.9% were females.

Since 1989, a total of 17 cases of horseshoe type appendix were reported in 11 countries, with 6 of these cases in China. To our knowledge, our case is thus only the 18th reported case worldwide, and the first 1 in France (Table 2, Fig. 4). Most affected patients were male (61.1%) and the mean age at diagnosis was 38.4 ± 18.2 years, ranging from 4 years old as the youngest case to 78 years old as the oldest. About 27.8% of cases were diagnosed at 40 to 50 years old (Table 3, Fig. 5).

**Table 2:** Reported cases of horseshoe appendix.

Author, year	Country	Sex, age	Diagnosis	Anatomical location	Operation	Reference
Mesko (1989)	USA	♂, 33 yr	Sigmoid diverticulitis	Unclear	Appendectomy	^[8]^
Dong (1994)	China	♂, 46 yr	Bowel occlusion	Cecum–cecum	Appendectomy + enterotomy	^[20]^
DasGupta (1999)	England	♂, 48 yr	Cecal perforation	Cecum–cecum	Appendectomy	^[9]^
Li and Yu (2000)	China	♀, 30 yr	Appendicitis	Cecum–cecum	Appendectomy	^[21]^
Cai and Lin (2017)	China	♂, 56 yr	Appendicitis	Cecum–cecum	Appendectomy	^[22]^
Calotă (2010)	Romania	♀, 43 yr	Intestinal occlusion	Cecum–cecum	Appendectomy	^[23]^
Ninos (2010)	Greece	♀, 20 yr	B-cell non-Hodgkin lymphoma	Cecum–cecum	Appendectomy + chemical therapy	^[24]^
Dube, (2011)	South Africa	♂, 32 yr	Appendicitis	Cecum-hepatic flexure of the colon	Appendectomy	^[25]^
Li and Liu (2012)	China	♂, 46 yr	Appendicitis + bowel occlusion	Cecum–cecum	Appendectomy + enterotomy	^[26]^
Oruç (2013)	Turkiye	♀, 64 yr	Appendicitis	Cecum–cecum	Appendectomy	^[27]^
Bulut (2015)	Turkiye	♀, 52 yr	Appendicitis	Cecum–cecum	Appendectomy	^[14]^
Singh (2016)	India	♂, 4 yr	Appendicitis	Cecum–cecum	Appendectomy	^[7]^
Takabatake (2016)	Japan	♂, 78 yr	Adenoma in colon	Cecum–ascending colon	Laparoscopic ileocecal resection	^[28]^
Liu (2018)	China	♂, 22 yr	Pneumonia appendicular mass	Cecum–cecum	Appendectomy	^[29]^
Zhu (2019)	China	♀, 44 yr	Fecal appendicitis right ovarian cyst	Cecum–cecum	Appendectomy, oophorectomy and partial resection of the small intestine	^[30]^
Choi (2019)	South Korea	♂, 33 yr	Appendicitis	Cecum–cecum	Laparoscopic appendectomy	^[31]^
Jabi (2021)	Morocco	♀, 26 yr	Appendicitis	Cecum–cecum	Appendectomy	^[32]^
Our case (2023)	France	♂, 15 yr	Appendicitis	Cecum–cecum	Laparoscopic appendectomy	This paper

**Table 3:** Descriptive statistics on age variable in horseshoe appendix cases.

		Total (N = 18)
Age	N	18
	Missing	0
	Mean (SD)	38.4 (18.2)
	Min; max	4.0; 78.0
Class of age	N	18
	Missing	0
	≤20	3 (16.7%)
	>20 to 30	3 (16.7%)
	>30 to 40	3 (16.7%)
	>40 to 50	5 (27.8%)
	>50	4 (22.2%)

### 3.3. Patient illness history

The medical history of these patients was documented for only 5 cases: 1 patient had generalized peritonitis caused by a perforated ulcer on the anterior wall of the superior part of duodenum, near the pylorus,^[23]^ 1 patient had frequent episodes of atypical lower abdominal discomfort for the last 2 years and had high grade B-cell non-Hodgkin lymphoma,^[24]^ 1 patient had tubulovillous adenoma,^[28]^ 1 patient had pneumonia and had been also treated for diabetes for 5 years,^[29]^ and another patient suffering from endometriosis had an 8-year history of repeated lower abdominal pain that appeared in the first 1 to 2 days of menstruation and disappeared after the end of menstruation.^[30]^

### 3.4. Clinical presentation

Although symptoms have varied from 1 case to another, most cases manifested abdominal pain radiated at the right iliac fossa (n = 10/18 cases),^[7,9,14,23,24,27,30–32]^ accompanied with tenderness (n = 9/18 cases),^[7,9,14,24,27,29–32]^ vomiting (n = 8/18 cases),^[7,9,23,29–32]^ nausea (n = 5/18 cases),^[14,27,29–31]^ and fever (n = 5/18).^[7,9,14,29,32]^ Moreover, there were few cases of loss of appetite (n = 3/18 cases),^[14,27,30]^ diarrhea (n = 1/18 cases),^[9]^ constipation (n = 1/18 cases),^[23]^ positive fecal occult blood test (n = 1/18 cases),^[28]^ general malaise (n = 1/18 cases) and cough with yellow sputum (n = 1/18 cases),^[29]^ and ketoacidosis (n = 1/18 cases).^[32]^ Overall, symptoms varied from 1 day to 2 weeks, and only 1 case involved immediate hospitalization.^[28]^ Regarding laboratory blood tests, there were 8 cases of leukocytosis,^[7,9,24,29–32]^ and 3 cases of elevated CRP levels (>10 mg/L) indicating inflammation.^[30,32]^

### 3.5. Diagnostic approaches

Half of the cases were diagnosed as appendicitis (n = 9/18) whereas in the rest, horseshoe appendicitis was incidentally found in patients with adenoma in the colon (n = 1/18), bowel occlusion (n = 2/18), B-cell non-Hodgkin lymphoma (n = 1/18), caecal perforation (n = 1/18), fecal appendicitis and right ovarian cyst (n = 1/18), intestinal occlusion (n = 1/18), pneumonia and appendicular mass (n = 1/18), and sigmoid diverticulitis (n = 1/18). The most affected anatomical location was cecum-cecum (83.3%). Cecum-ascending colon and cecum-hepatic flexure of the colon were also observed in 2 individual cases. Only 1 case did not have a clear anatomical location (Table 2).

Among imaging examinations, CT scans^[9,28–31]^ and ultrasonography^[7,14,27,29,32]^ were used in 6 cases. Moreover, chest-abdominal radiology was used in 3 cases^[14,23,27]^ whereas colonoscopy was used in only 1 case.^[28]^ Out of 18 cases, only 3 imaging results confirmed the diagnosis of AA,^[31,32]^ however, none of them revealed the horseshoe appearance of the inflamed appendix.

### 3.6. Treatment and prognosis

All patients received appendectomy (Table 2), in which 9 cases detected horseshoe appendix via surgical exploration,^[14,23,24,27,28,30–32]^ and 5 cases utilized laparotomy.^[14,28,30,31]^ Two cases involved conservative management with antibiotics for 6 weeks^[9]^ or 4 months^[7]^ before interval appendicectomy. Moreover, intra operative findings revealed 3 cases of perforation,^[9,14]^ 1 case where the appendix was connected to the ascending colon,^[28]^ and 1 case of enlarged mesenteric lymph nodes.^[24]^ Double appendicectomy was performed in 3 cases,^[14,24,29]^ whereas retrograde appendectomy was performed in 2 cases,^[27,31]^ and adhesiolysis ligature in 1 case.^[23]^

Macroscopic findings showed the appendix connected to the back side of the mass, inserting along the appendiceal orifice. Moreover, the mucosa and submucosa were continuous from the appendiceal orifice in the cecum to the other orifice in the ascending colon with a seamless muscular layer. There was no evidence of inflammation or malignancy, and pathologically, the appendix was normal. However, there was a type 1 tumor on the orifice in the ascending colon, which was pathologically diagnosed as a tubulovillous adenoma with moderate atypia, along with an appendiceal extension and reaching the adenoma of the ascending colon.^[28]^

Out of 18 cases, 8 patients recovered uneventfully,^[7,9,24,27–31]^ whereas 1 patient had paralytic ileus,^[28]^ and another was treated for ketoacidosis.^[32]^ The rest were undocumented.

### 3.7. Horseshoe appendix vs other duplex appendix types

According to a recent systematic review by Nageswaran et al published in 2018, a total of 141 cases of appendiceal duplication were identified, in which the male/female ratio was 1.4:1 and the median age 20 years (ranging from a fetus to 69 years old). Most cases were categorized by the Cave–Wallbridge classification: There were 22 Type A, 8 Type B1, 46 Type B2, and 10 Type C cases. Concerning the horseshoe type appendix, only 6 cases were reported.^[33]^ This illustrates the rarity of this type of duplex appendix and thus, a careful examination of the cecal pole and possible exploration of the retrocecal space for appendiceal duplication should be considered whenever there are convincing clinical or radiological signs of appendicitis.

## 4. Conclusions

In summary, horseshoe type appendix is a rare form of duplicated appendiceal anomaly that can be discovered in children or adults. Most commonly presenting with lower right-side pain, as seen in other types of appendicitis, and are incidentally detected via surgical exploration. Lab imaging such as radiology and CT scans helped confirming the diagnosis of AA in few cases, but none of them showed the horseshoe appearance of the inflamed appendix. Based on the 18 cases reviewed, treatment varied from 1 patient to another but all required appendicectomy. Therefore, the inspection of double appendices such as the “horseshoe” type should be considered during appendectomy despite their rarity, as their misdiagnosis and lack of adapted treatment may lead to serious health consequences and medical complications. Bacteriological analysis of the perforated appendix provides physicians with information for better antibiotics management and consequently improve patients’ outcomes.

## Acknowledgments

Authors would like to thank Dr Frédérique Bois-Langlois, a radiologist at Hôpital Privé Guillaume de Varye, 210 Route de Vouzeron, 18230 Saint-Doulchard, for radiological images, and Dr Charlotte Bothorel, an anatomical pathologist at Unilabs SIPATH, 18 Avenue Lénoard De Vinci, 63000 Clermont Ferrand, for histological images. Authors would also like to thank Asmaa Missoum (MultiHealth Group) for original draft writing and Mickael Borne (MultiHealth Group) for data curation.

## Author contributions

**Conceptualization:** Hannah Pflieger.

**Data curation:** Christine Tchikladzé-mérand.

**Supervision:** Hannah Pflieger.

**Writing – review & editing:** Hannah Pflieger.
